# Conjugated Linoleic Acid Production by Bifidobacteria: Screening, Kinetic, and Composition

**DOI:** 10.1155/2016/8654317

**Published:** 2016-06-27

**Authors:** Stefano Raimondi, Alberto Amaretti, Alan Leonardi, Andrea Quartieri, Caterina Gozzoli, Maddalena Rossi

**Affiliations:** Department of Life Sciences, University of Modena and Reggio Emilia, Via Campi 103, 41125 Modena, Italy

## Abstract

Conjugated linoleic acids (CLA) are positional and geometric isomers of linoleic acid involved in a number of health aspects. In humans, CLA production is performed by gut microbiota, including some species of potential probiotic bifidobacteria. 128 strains of 31* Bifidobacterium* species were screened with a spectrophotometric assay to identify novel CLA producers. Most species were nonproducers, while producers belonged to* B. breve* and* B. pseudocatenulatum*. GC-MS revealed that CLA producer strains yielded 9*cis*,11*trans*-CLA and 9*trans*,11*trans*-CLA, without any production of other isomers. Hydroxylated forms of LA were absent in producer strains, suggesting that the myosin-cross-reactive antigen (MCRA) protein that exerts hydratase activity is not involved in LA isomerization. Moreover, both CLA producer and nonproducer species bear a MCRA homologue. The strain* B. breve *WC 0421 was the best CLA producer, converting LA into 68.8% 9*cis*,11*trans*-CLA and 25.1% 9*trans*,11*trans*-CLA. Production occurred mostly during the lag and the exponential phase. For the first time, production and incorporation of CLA in biomass were assessed.* B. breve* WC 0421 stored CLA in the form of free fatty acids, without changing the composition of the esterified fatty acids, which mainly occurred in the plasmatic membrane.

## 1. Introduction

Conjugated linoleic acids (CLA) are a group of positional and geometric isomers of linoleic acid (LA,* cis9*,* cis12*-C18:2) that have conjugated double bonds. Diverse CLA isomers have been identified with different position (ranging from Δ7, Δ9 to Δ12, Δ14) and cis/trans geometry. 9*cis*,11*trans*-CLA and 10*trans*,12*cis*-CLA are the major isomers occurring in human diet, the former being contained mostly in ruminant meat and dairy products and the latter in hydrogenated oils. Considerable research has been directed toward understanding the production and the physiological effects of CLA and there is increasing evidence of their involvement in a number of health aspects. Potential health-promoting properties of CLA include anticarcinogenesis, antiatherosclerosis, antidiabetes, antiobesity, antiallergy activities, and enhancement of immune functions [1].

CLA are intermediates of the biohydrogenation of linoleic acid (LA) and unsaturated fatty acids, a microbial process by which the fatty acids released by lipolytic enzymes undergo the progressive saturation. Microbial isomerization of LA into CLA was firstly described in the gut of ruminants, but diverse isolates from human feces have been demonstrated to take part in LA biohydrogenation, indicating that production of CLA and 11*trans*-C18:1 (vaccenic acid) occurs in the human gut as well [2–5]. In fact, humans excrete some linoleic acid (LA) with feces [6], suggesting that this substrate is available for microbial production of CLA within the colon. Several colonic species belonging to Cluster IV, Cluster XIVa, and* Bifidobacterium* genus are involved in LA transformation toward CLA, including 9*cis*,11*trans*-CLA and vaccenic acid.

Some species of bifidobacteria, natural colonizers of the gut, are capable to transform LA into 9*cis*,11*trans*-CLA and into lower amounts of 9*trans*,11*trans*-CLA and 10*trans*,12*cis*-CLA. Previous studies suggested that the MCRA protein of bifidobacteria may be involved in isomerization of LA to CLA, through hydration of unsaturated fatty acids, for example, converting palmitoleic, oleic, and linoleic acids to the corresponding 10-hydroxy fatty acids [7, 8]. However, more recent evidences excluded the role of bifidobacterial MCRA in CLA production [9].

The ability of various bifidobacteria to produce 9*cis*,11*trans*-CLA raised the question as to whether CLA production may be regarded as one of the mechanisms by which bifidobacteria exert some of their beneficial properties [10]. In fact, it is known that commensal bifidobacteria exert a number of beneficial health effects through a variety of different mechanisms.

For this reason, they are increasingly being used in functional foods and pharmaceutical products and are generally regarded as probiotics [11–13]. The present study aimed to identify novel CLA producers among a wide range of* Bifidobacterium* species and strains and to explore unedited aspects of CLA accumulation, such as the effect of LA and CLA in microbial lipid composition and the accumulation of hydroxylated forms of linoleic acid, ascribable to MCRA activity.

## 2. Materials and Methods

### 2.1. Chemicals, Strains, and Culture Conditions

All chemicals were purchased from Sigma Aldrich (Steinheim Germany) unless otherwise stated. 128 strains of* Bifidobacterium* (Table 1) were obtained from ATCC or from our culture collection (WC-labeled strains). Bifidobacteria were cultured in lactobacilli deMan-Rogosa-Sharpe broth (Becton Dickinson, Franklin Lakes, NJ, USA) supplemented with 0.5 g/L L-cysteine·HCl (hereinafter called MRS), at 37°C in an anaerobic cabinet (Forma Scientific, Marietta, OH, USA) under N_2_ 85%, CO_2_ 10%, and H_2_ 5% atmosphere.

The analysis of the biotransformation of linoleic acid (LA) into conjugated linoleic acid (CLA) was carried out in MRS broth supplemented with 0.5 g/L LA (hereinafter called MRS-LA). A solution of 40 mg/mL LA in 20 g/L Tween 80 was prepared, filter sterilized (cellulose acetate, 0.22 *μ*m; Millipore, Billerica, MA, USA), and added to the sterile MRS in proper amount to obtain 0.5 g/L LA. Bifidobacteria from 16 h MRS cultures were inoculated (10% v/v) into MRS-LA and incubated at 37°C for 48 h.

### 2.2. Kinetic of CLA Production and Bioreactor Operation

Controlled-pH batch cultures were carried out in laboratory-scale fermenters (Sixfors, Infors, Bottmingen, Switzerland) with 500 mL of MRS or MRS-LA medium. The bioreactor was inoculated with 50 mL of a 24 h seed culture grown in MRS or MRS-LA medium, respectively. To investigate CLA production during stationary phase, LA was added to MRS cultures after 24 h of growth. Temperature was kept at 37°C, stirring at 200 rpm, and pH was maintained constant at 5.5 through the automatic addition of 4 M NaOH. The bioreactor was kept under nitrogen overpressure to maintain anaerobiosis conditions. The culture was periodically sampled to monitor bacterial growth, glucose consumption, and the concentration of LA and CLA in the broth. The biomass/substrate yield (*Y*_X/S_) was expressed as g of dry biomass g^−1^ consumed glucose. During the mid-exponential growth phase and at the entrance of the stationary phase, 200 mL of the culture were collected in order to separate the biomass and perform on it the analysis of lipid fraction.

### 2.3. Spectrophotometric Analysis of CLA in the Supernatant

In order to screen all the bifidobacteria for the capability to produce CLA, a rapid spectrophotometric method was used, based on UV absorption of conjugated double bonds [14]. The culture sample was centrifuged (13,000 ×g for 5 min at 4°C) and 1 mL of supernatant was mixed with 2 mL of isopropanol. After the addition of 1.5 mL of hexane, the sample was thoroughly vortexed in order to extract the lipids and then allowed to stand 5 min. The hexane layer was collected and the absorbance was measured at 233 nm (*A*_233_).

### 2.4. Extraction of Lipids and Preparation of Fatty Acyl-Methyl-Esters

To extract cell-associated lipids, the biomass of 200 mL of culture was harvested by centrifugation (9,000 ×g for 10 min at 4°C), washed with water, frozen at −80°C, and lyophilized (Heto Lyolab 3000, Allerød, Denmark). Lyophilized biomass was mixed (2% w/v) with a 2 : 1 (v/v) chloroform : methanol solution and shaken at r.t. for 16 h. The extract was filtered through celite and anhydrous Na_2_SO_4_, solvents were removed using a rotavapor apparatus, and lipids were weighed. To determine the amount of LA and CLA in the supernatant, 3 mL was thoroughly vortexed with 3 mL of ethyl acetate. The organic phase was separated and anhydrified; then the solvent was removed.

The free fatty acids (FFAs) and the esterified fatty acids (EFAs), such as glycerides and phospholipids, were transformed into the corresponding methyl-esters (FAMEs) to be analyzed by GC-MS. The FFAs contained in the lipid extract of the biomass or the supernatant were methylated through diazomethane reaction [15]. The extract was dissolved in the ethyl ether solution of diazomethane, freshly prepared from Diazald, and incubated at r.t. for 10 min. Once the solvent had evaporated, FAMEs were dissolved in 1 mL of ethyl acetate containing 0.5 mg/mL methyl undecanoate as internal standard and analyzed.

The EFAs contained in the lipid extract of the biomass were transesterified through alkali-catalyzed reaction [16]. The lipid extract was dissolved in 2 mL of ethyl ether containing 1 mg/mL triundecanoin as internal standard. 50 *μ*L of 3.3 M sodium methoxide (methanol solution) was added to the reaction mixture and the sample was homogenized. After 10 min incubation at r.t., the reaction was stopped with 30 *μ*L of acetic acid.

### 2.5. GC-MS Analysis

The organic phases containing FAMEs were injected (0.5 *μ*L) into a GC apparatus (HP5890 Series II, Agilent, Waldbronn, Germany) equipped with the column (100 m × 0.25 mm, 0.25 *μ*m) CP-Select FAME (Varian, Palo Alto, CA, USA). The injector was kept at 270°C. To determine the whole profile of FAs, the oven temperature ramped from 135°C to 250°C (2.5°C/min) and was maintained at 250°C for 23 min. The same program was followed, starting from 160°C, when only LA and CLA had to be determined. Elution was performed with high-purity helium, with constant column head pressure of 200 kPa. Qualitative analyses of FAMEs were performed with a MS quadrupole detector (HP5972, Agilent). Analytes were identified by comparison with standards (O5632, Sigma Aldrich) and by analysis of their fragmentation patterns (EI, 70 eV) with the mass spectrum library NIST 2005 (Gatesburg, USA). Quantitative analyses were performed with a flame ionization detector held at 300°C.

### 2.6. Alignment Analysis of Putative* Bifidobacterium* MCRA Genes and PCR Amplification

In order to verify the presence of homologues of MCRA from* B. breve* NCIMB 702258 (Protein ID ADY18551.1) a search was performed among all the species of* Bifidobacterium*, using the tblastn program provided by the National Center of Biotechnology Information (http://www.ncbi.nlm.nih.gov/). The available sequences of putative MCRA gene of* Bifidobacterium* spp. were aligned using the Clustal omega program (http://www.ebi.ac.uk/). To amplify the conserved region of MCRA genes, the primers MCRAf (5′-GCGGSCGCGARATGGACAA-3′) and MCRAr (5′-CCGCCGTTGGTGATGAACAC-3′) were designed. These primers anneal with the gene sequence of* B. breve* NCIMB 702258 (GenBank accession HQ593838H) at positions 236–255 and 958–978 and generate amplicons of 742 bp. Amplification was performed with the following program: 4 min at 94°C; 30 cycles of 30 s at 94°C; 30 s at 58°C; 1 min at 72°C; 1 min at 72°C.

### 2.7. Statistical Analysis

All values are described by the means and the standard deviation of three separate experiments. Comparisons were performed with one-way ANOVA followed by Tukey* post hoc* comparisons. Differences were considered statistically significant for *P* < 0.05. Statistical analysis was done using GraphPad Prism 4.0 (GraphPad Software, San Diego, CA, USA).

## 3. Results

### 3.1. Spectrophotometric Screening of Conjugated Linoleic Acid Production

127* Bifidobacterium* strains belonging to 31 different species or subspecies (Table 1) were cultured in MRS-LA medium, containing 0.5 g/L of free LA. With the exception of 3 strains belonging to the species* B. coryneforme*,* B. gallinarum*, and* B. saeculare*, unable to grow in presence of LA, all bifidobacteria grew in MRS-LA achieving OD_600_ ranging between 1.1 and 5.7 after 24 h (data not shown).

**Figure 1:**
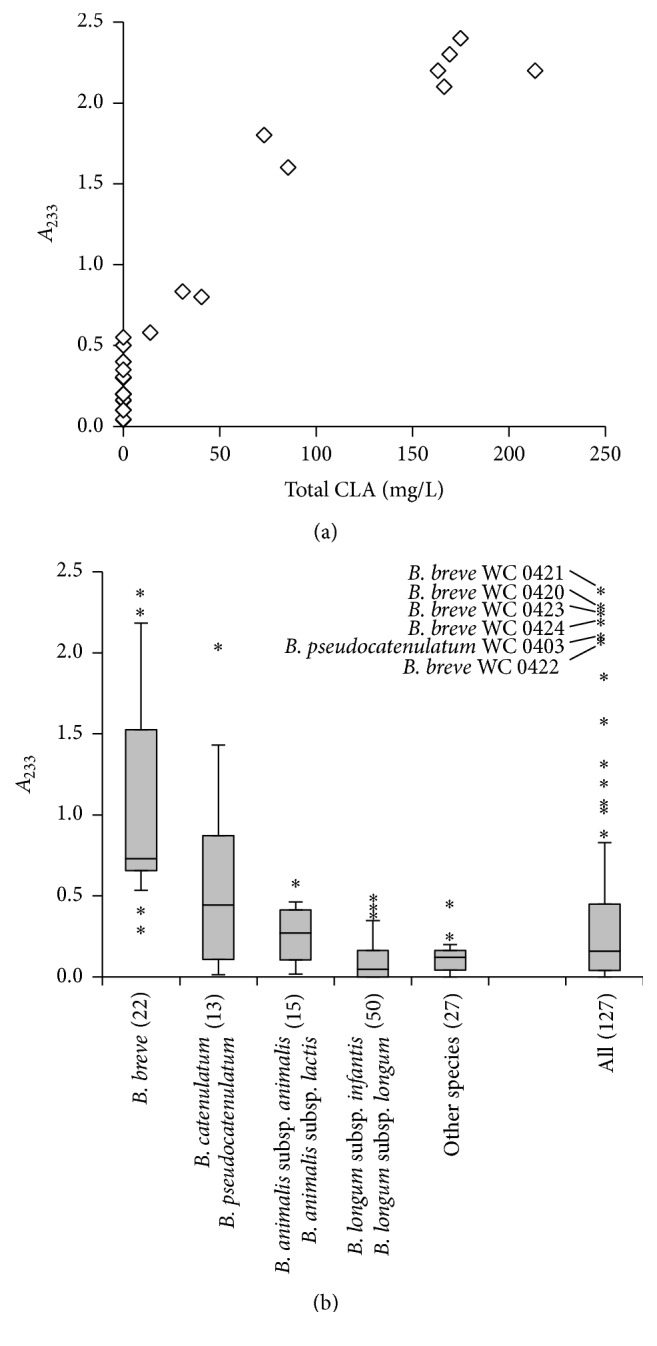
UV absorbance (*A*_233_) of the lipophilic extracts that were obtained from the supernatants of 24 h MRS-LA cultures of bifidobacteria. (a) Relationship between *A*_233_ and total CLA concentration in the supernatant in a selection of 34 strains. (b) Screening of 127* Bifidobacterium* strains. Boxes represent the range between 25th and 75th percentiles and the line inside represents the median. Whiskers denote the range between 10th and 90th percentiles and asterisks represent outlier values. Strains numbers are in brackets.

To obtain a preliminary estimation of CLA production, the supernatant of 24 h cultures in MRS-LA was extracted, and the UV absorbance by conjugated double bonds was measured at *λ* = 233 nm (*A*_233_). *A*_233_ ranged between 0 and 2.37 a.u., with the vast majority of the strains (>75%) yielding an extract with absorbance lower than 0.5 a.u. With the aim to validate the spectrophotometric assay, total CLA were quantified by GC-MS in the supernatant of 34 strains belonging to the species* B. animalis* subsp.* lactis*,* B. bifidum*,* B. breve*,* B. catenulatum*,* B. longum* subsp.* infantis*,* B. longum* subsp.* longum*, and* B. pseudocatenulatum*, covering the whole *A*_233_ range. The dot dispersion of *A*_233_ against CLA production revealed that CLA production can be excluded for *A*_233_ < 0.5 (Figure 1(a)). Above this value, higher absorbance roughly corresponded to higher CLA concentration, although a direct relationship could not be established (*R* ^2^ = 0.87).

All the strains belonging to the species* B. adolescentis, B. angulatum, B. bifidum, B. boum, B. choerinum, B. cuniculi, B. dentium, B. gallicum, B. indicum, B. magnum, B. merycicum, B. minimum, B. pullorum, B. pseudolongum, B. psychraerophylum, B. ruminantium, B. scardovii, B. subtile*, and* B. thermophilum* and most of the strains belonging to* B. animalis* subsp.* animalis, B. animalis* subsp.* lactis, B. longum* subsp.* infantis, B. longum* subsp.* longum, *and* B. longum* subsp.* suis* fell into the group of CLA producers (Figure 1(b)).

The species* B. breve* and the group of* B. catenulatum* and* B. pseudocatenulatum* were the highest CLA producers, even though wide variability was observed. The extracts of* B. breve* presented the highest *A*_233_ (mean = 1.08, median = 0.73), followed by those of* B. catenulatum* and* B. pseudocatenulatum* (mean = 0.62, median = 0.45). All the strains with *A*_233_ higher than the 90th percentile belonged to the species* B. breve* and* B. pseudocatenulatum* (Figure 1(b)). In particular, one strain of* B. pseudocatenulatum* and five of* B. breve* presented *A*_233_ higher than 2.0 a.u. and were selected for deeper investigation of CLA production.

### 3.2. Search of MCRA Gene in* Bifidobacterium* Species

A tblastn search was performed for all the species of* Bifidobacterium* reported in Table 1, in order to verify the presence of a homologue of MCRA from* B. breve* NCIMB 702258 (Protein ID ADY18551.1). Of the 31 species, 14 bear a gene encoding a protein with sequence identity from 55 to 94%:* B. adolescentis*,* B. angulatum*,* B. animalis* subsp.* animalis*,* B. animalis* subsp.* lactis*,* B. bifidum*,* B. breve*,* B. catenulatum*,* B. dentium*,* B. longum* subsp.* infantis*,* B. longum* subsp.* longum*,* B. pseudocatenulatum*,* B. pseudolongum*,* B. scardovii*, and* B. thermophilum*. All the 34 strains that were selected for the GC quantification of CLA were positive to MCRA-targeted PCR, yielding an amplicon of approx. 750 bp (Table 1).

### 3.3. CLA Production by Selected Strains

Yields and the composition of CLA were determined in the supernatants of* B. breve* WC 0420,* B. pseudocatenulatum* WC 0403,* B. breve* WC 0422,* B. breve* WC 0424,* B. breve* WC 0423, and* B. breve* WC 0421, after 48 h of growth in MRS-LA (Table 2). Conversion of LA into CLA ranged between 13.2 and 88.1%.* B. breve* WC 0421 and* B. breve* WC 0423 were the most efficient CLA producers, with yields of 88.1 and 87.6%, respectively. 9*cis*,11*trans*-CLA and 9*trans*,11*trans*-CLA were the sole CLA isomers occurring in supernatants. The former was the major product of LA isomerization, accounting for 13.2 to 81.1% of LA conversion and for 77 to 100% of total CLA isomers. The latter was generally found in lower amount, never exceeding the 7.3% of LA conversion and the 23% of total CLA. The ratio 9*cis*,11*trans*-CLA : 9*trans*,11*trans*-CLA ranged between 3.4 and 11.6 and increased augmenting the total amount of CLA produced. None of these strains produced hydroxylated forms of LA.

### 3.4. Kinetic of LA Isomerization into CLA by* B. breve* WC 0421

**Figure 2:**
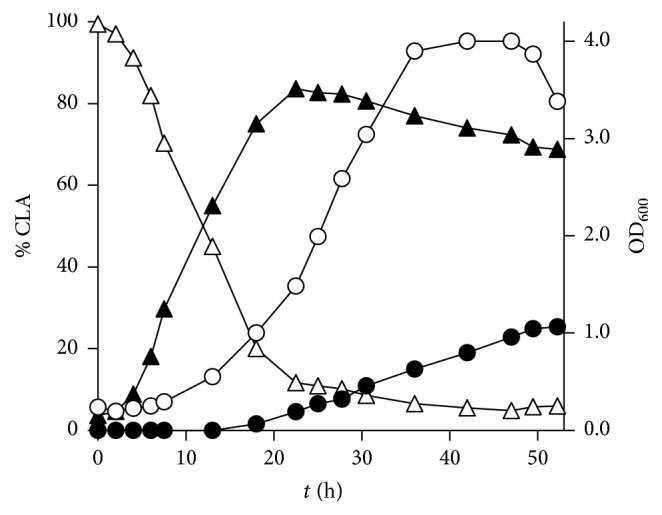
Time-course of growth and CLA production by* B. breve* WC 0421 in MRS medium containing 0.5 g/L LA. ◯: turbidity (OD_600_), ∆: LA (%), ▲: 9*cis*,11*trans*-CLA (%), and ●:* trans*9,*trans*11-CLA (%). The results are representative of the three independent experiments.

Constant-pH batch cultures of* B. breve* WC 0421 were carried out in MRS and MRS-LA. The presence of 0.5 g/L LA caused an extension of the lag-phase of approx. 6 h, slowed down the specific growth rate (0.33 and 0.11 h^−1^ in MRS and MRS-LA, resp.), and delayed the entrance into stationary phase of 2 h. The biomass/glucose yield was lower in MRS-LA than in MRS (8 and 13%, resp.), whereas the percentage lipid/biomass was higher in the medium containing LA (7.5% and 2.5%, resp.). The isomerization of LA into CLA occurred mostly during the lag and the exponential phases (Figure 2). After 24 h of fermentation, during the exponential phase, the transformation of LA resulted in the production of 9*cis*,11*trans*-CLA (83.6%) and, at a minor extent, of* trans*9,*trans*11-CLA (4.5%). During the late exponential and stationary phases, LA isomerization proceeded slowly and 9*trans*,11*trans*-CLA progressively accumulated at the expenses of 9*cis*,11*trans*-CLA. Altogether, the conversion yield of LA into CLA was 94.3% after 52 h. In particular, conversion yields into 9*cis*,11*trans*-CLA and 9*trans*,11*trans*-CLA were 68.8 and 25.1%, respectively. All other CLA isomers were always negligible throughout the fermentation.

When 0.5 g/L LA was added to MRS cultures at the entrance into the stationary phase, the isomerization occurred at minor extent, resulting in production of the sole 9*cis*,11*trans*-CLA isomer with a conversion of 6.1% after 48 h incubation.

### 3.5. Lipid Composition of the Biomass

The profile of free and esterified fatty acids (FFA and EFA) within the lipid fraction of* B. breve* WC 0421 biomass was investigated (Table 3). In MRS, lipids were dominated by EFA (*ca* 93% of total lipids), while FFA were found in low amounts (≤8%). At the exponential and stationary phases, oleic acid was the most abundant among both EFA and FFA, occurring for more than 40%. Palmitic and myristic acids and palmitic and stearic acids represented the other major fatty acids among EFA and FFA, respectively.

During the cultivation in MRS-LA,* B. breve* WC 0421 accumulated a high amount of FFA, which accounted for* ca* 75% of total lipids at the stationary phase. LA occurred in low amount (<4%) among both EFA and FFA. 9*cis*,11*trans*-CLA dominated the profile of FFA, always accounting for more than 56%. The composition of EFA was similar in MRS and MRS-LA.

The supplementation of 0.5 g/L LA at the entrance into the stationary phase did not cause accumulation of relevant amounts of CLA as free or esterified fatty acid in the microbial biomass. 24 h after LA addition, EFA composition remained unchanged, while FFA increased up to 70% of the lipid extract. In this case, FFA were mostly composed of LA (77%), whereas CLA altogether accounted for* ca *4%.

## 4. Discussion

The present study aims to gain further insight into CLA production by bifidobacteria, since the capability of certain strains to convert LA into CLA could find application in the development of specific probiotic strains able to accelerate or improve* in vivo* conversion of LA into CLA.

*In vivo* studies supporting biological activity of CLA were initially performed using a mixture of CLA isomers, while purified isomers have been used more recently, supporting the evidence that different isomers have distinct effect on the major health targets, such as tumorigenesis and lipid metabolism [1]. However, most of commercial formulations of CLA still contain both major isomers, whose biological activity is mediated by diverse signaling pathways. 9*cis*,11*trans*-CLA isomer exerts an enhancing effect on the nuclear transmission of PPAR*γ*, a master regulator of adipocyte differentiation, acting as a stimulator of adiponectin secretion [17]. This mechanism can to some extent explain the antihypertensive, antihyperlipidemic, antiarteriosclerotic, anticarcinogenic, and antidiabetic effects mediated by this CLA [18]. Conversely,* trans*10,*cis*12-CLA increases lipolysis and has the function of diminishing the synthesis of fatty acids [19] but also presents a linkage with proatherogenic effects, insulin resistance, and inflammation [20].

The perspective to exploit specifically selected probiotic bifidobacteria to produce a definite CLA isomer, such as the 9*cis*,11*trans*-CLA, is quite attractive. The data herein presented demonstrate that selected strains of bifidobacteria specifically convert linoleic acid (LA) into 9*cis*,11*trans*-CLA isomer, whereas 10*trans*,12*cis*-CLA, for which potential harmful biological activities are still debated, is not produced by the tested strains.

The screening of CLA production among the major species of bifidobacteria demonstrated that the most efficient CLA producers belong to the species* B. breve* and* B. pseudocatenulatum*. Consistently,* B. breve* strains have been previously identified among the best CLA producers, whereas low or negligible activity was found in the strains belonging to* B. adolescentis*,* B. angulatum*,* B. bifidum,* and* B. infantis* [2, 9, 21]. Unlike previous studies that indicated* B. pseudocatenulatum* as almost incapable of LA transformation [2], our data revealed that some strains of* B. pseudocatenulatum* can efficiently accomplish the isomerization of LA.

The data herein presented are in agreement with observations by O'Connell et al. [9], which excluded that the product of MCRA gene is involved in LA isomerization to CLA. In the present study, all the best producers of CLA, belonging to the species* B. breve* and* B. pseudocatenulatum*, were positive to this gene, yet no hydroxylated forms of LA were detected in their supernatants or in the biomass of* B. breve* WC 0421. A further clue hinting that MCRA is not involved in CLA production is the fact that both MCRA-targeted PCR and BLAST search for the gene revealed the presence of a homologue in CLA producer and nonproducer species.

*B. breve* WC 0421 emerged as the most promising strain for production of 9*cis*,11*trans*-CLA isomer. Our results associate the production of* cis*9,*trans*11-CLA to the lag and early growth phases, whereas supplementation of LA into stationary phases resulted in negligible production of CLA. This result supports the hypothesis that transformation of LA in CLA can be a strategy of bacteria to detoxify unsaturated fatty acid that interfere with membrane lipids. These* in vitro* experiments have been carried out in batch, with a pure culture, on a rich medium, with condition very different from the intestinal ecosystem, where bifidobacteria represent only a few percent of a complex mixed population. However, bioproduction of CLA by supplemental probiotic bifidobacteria has been previously demonstrated [10]. For this reason,* B. breve* WC 0421 may represent a promising candidate for* in vivo* production of the specific health-promoting CLA isomer.

The attention of the scientific community has been focused on CLA production and absorption in the gastrointestinal tract. The analysis of fatty acid profile of biomass associated lipids revealed that when* B. breve* WC 0421 was cultured in presence of LA it accumulated CLA in the form of free fatty acids, without changing the composition of its esterified fatty acids, mainly occurring in the plasmatic membrane.

## 5. Conclusions

Bifidobacteria are one of the most relevant health-promoting indigenous species of the human gut microbiota, exerting beneficial positive effects through a variety of different mechanisms. Our study strengthened the perspectives on a more specific use of probiotics, where the intrinsic health-promoting properties of bifidobacteria can be associated with peculiar features, such as producing* in vivo* health-promoting CLA. Based on the current knowledge [2, 9, 21] and on the outcome of this study, selected strains of* Bifidobacterium*, mostly belonging to the species* B. breve*, can be exploited as probiotics for* in vivo* production of 9*cis*,11*trans*-CLA.

The assessment of production and incorporation of CLA in bifidobacterial biomass is a relevant result of our study. Since the host-bacteria interaction is mediated by several surface compounds, it is necessary to investigate the location of 9*cis*,11*trans*-CLA within bifidobacterial cells and to examine whether CLA enriched bifidobacteria exert a diverse biological effect than the corresponding CLA-free cells.

## Figures and Tables

**Table 1:** *Bifidobacterium* strains screened for CLA production, by measuring the absorbance of conjugate diene (*A*_233_) after 24 h growth in MRS-LA. *∗* indicates species bearing a gene homologue to MCRA from *B. breve* NCIMB 702258. The 34 strains that were selected for GC quantification of CLA and PCR amplification are underscored.

Species	Strains
*B. adolescentis* ^*∗*^	ATCC 15703, WC 9806
*B. angulatum* ^*∗*^	ATCC 27535
*B. animalis* subsp*. animalis* ^*∗*^	ATCC 25527, WC 0409, WC 9343
	WC 0410
*B. animalis* subsp*. lactis* ^*∗*^	ATCC 27536, WC 0411, WC 0412,
	WC 0413, WC 0414, WC 0432,
	WC 0455, WC 0459, WC 0460,
	WC 0469, WC 0471
*B. asteroides*	ATCC 25910
*B. bifidum* ^*∗*^	WC 0415, WC 0417, WC 0418
*B. boum*	ATCC 27917
*B. breve* ^*∗*^	WC 0420, WC 0421, WC 0422,
	WC 0423, WC 0424, WC 9436,
	WC 9437, WC 9445, WC 9445,
	WC 9446, WC 9449, WC 9491,
	WC 9492, WC 9493, WC 9495,
	WC 9498, WC 9499, WC 9500,
	WC 9501, WC 9504, WC 9505,
	WC 9508
*B. catenulatum* ^*∗*^	ATCC 27539, WC 0458, WC 0467
*B. choerinum*	ATCC 27686
*B. coryneforme*	ATCC 25911
*B. cuniculi*	ATCC 27916
*B. dentium* ^*∗*^	ATCC 27534
*B. gallicum*	ATCC 49850
*B. gallinarum*	ATCC 33777
*B. indicum*	ATCC 25912
*B. longum* subsp*. infantis* ^*∗*^	ATCC 15697, WC 0433,
	WC 0434
*B. longum* subsp*. longum* ^*∗*^	ATCC 15707, WC 0435, WC 0436,
	WC 0437, WC 0438, WC 0439,
	WC 0440, WC 0441, WC 0442,
	WC 0443, WC 0444, WC 0473,
	WC 9711, WC 9712, WC 9717,
	WC 9718, WC 9721, WC 9722,
	WC 9724, WC 9741, WC 9742,
	WC 9743, WC 9745, WC 9746,
	WC 9747, WC 9748, WC 9749,
	WC 9751, WC 9752, WC 9753,
	WC 9754, WC 9756, WC 9757,
	WC 9758, WC 9759, WC 9760,
	WC 9761, WC 9762, WC 9763,
	WC 9765, WC 9766, WC 9767,
	WC 9768, WC 9769, WC 9770,
	WC 9772, WC 9773
*B. longum* subsp*. suis*	ATCC 27533
*B. magnum*	ATCC 27540
*B. merycicum*	ATCC 49391
*B. minimum*	ATCC 27538
*B. pullorum*	ATCC 27685
*B. pseudocatenulatum* ^*∗*^	ATCC 27919, WC 0400, WC 0401,
	WC 0402, WC 0403, WC 0404,
	WC 0405, WC 0407, WC 0408,
	WC 9359
*B. pseudolongum* ^*∗*^	ATCC 25526
*B. psychraerophilum*	NCIMB 13940
*B. ruminantium*	ATCC 49390
*B. saeculare*	ATCC 49392
*B. scardovii* ^*∗*^	ATCC BAA-773
*B. subtile*	ATCC 27683
*B. thermophilum* ^*∗*^	ATCC 25525

**Table 2:** Residual LA (%) and conversion yield into 9*cis*,11*trans*-CLA, 9*trans*,11*trans*-CLA, and total CLA (%) by bifidobacteria cultured for 48 h in MRS-LA medium.

	LA	9*cis*,11*trans*-CLA	9*trans*,11*trans*-CLA	Total CLA
*B. breve* WC 0420	86.8^a^	13.2^a^	0.0^a^	13.2^a^
*B. pseudocatenulatum* WC 0403	81.5^a^	14.3^a^	4.2^b^	18.5^b^
*B. breve* WC 0422	58.2^b^	37.2^b^	4.7^b^	41.8^c^
*B. breve* WC 0424	22.7^c^	70.2^c^	7.0^c^	77.3^d^
*B. breve* WC 0423	12.4^d^	80.3^d^	7.3^c^	87.6^e^
*B. breve* WC 0421	11.9^d^	81.1^d^	7.0^c^	88.1^e^

Values are means, *n* = 3; SD always < 5%. Means in a column with different superscripts significantly differ (*P* < 0.05).

**Table 3:** Relative composition (%) of esterified and free fatty acids (EFAs and FFAs, resp.) in the biomass of *B. breve* WC 0421 at the exponential and the stationary phase in MRS and MRS-LA.

			MRS		MRS-LA	
			Exponential	Stationary	Exponential	Stationary
EFA	EFA/total FA		93^a^	92^a^	26^b^	25^b^
	C10		0.8^a^	1.7^b^	1.2^a^	1.7^b^
	C12		2.8^a^	4.2^b^	3.9^b^	4.2^b^
	C14		8.6^a^	16.6^b^	13.7^b^	15.7^b^
	C16		17.4	21.5	18.9	22
	C16:1		1.1	1.9	1.9	2
	C17		0.9	0.5	0	0.4
	C18		4.2^a^	3.1^a^	0.0^b^	1.7^b^
	C18:1		55.8^a^	41.3^b^	50.9^a^	43.9^b^
	C18:2	LA	2.1	3.2	3.7	3.1
	C18:2	9*cis*,11*trans*-CLA	1.6	1.2	0.9	1
	C18:2	9*trans*,11*trans*-CLA	3.6	3.9	5	4
	C20		0.3	0.3	0	0.1
	C22		0.8	0.5	0	0.4

FFA	FFA/total FA		7^a^	8^a^	74^b^	75^b^
	C10		0	0	0	0
	C12		0	0	0	0
	C14		3.2^a^	12.7^b^	1.2^a^	0.6^a^
	C16		23.6^a^	22.0^a^	5.7^b^	2.4^b^
	C16:1		0	0	0	0
	C17		3.1^a^	1.6^a^	0.6^b^	0.3^b^
	C18		10.4^a^	6.8^a^	2.5^b^	1.2^b^
	C18:1		47.0^a^	46.2^a^	13.9^b^	8.3^b^
	C18:2	LA	0.0^a^	0.0^a^	1.7^b^	2.8^b^
	C18:2	9*cis*,11*trans*-CLA	2.1^a^	2.7^a^	58.6^b^	56.6^b^
	C18:2	9*trans*,11*trans*-CLA	3.3^a^	7.1^a^	15.2^b^	27.0^c^
	C20		4	0.9	0.2	0.4
	C22		3.2	0	0.5	0.3

Values are means, *n* = 3; SD always < 5%. Means in a row with different superscripts significantly differ (*P* < 0.05).
